# You & Me: Test and Treat study protocol for promoting COVID-19 test and treatment access to underserved populations

**DOI:** 10.1186/s12889-023-16960-6

**Published:** 2023-10-28

**Authors:** Emily M. D’Agostino, Lauren M. Rosenberg, Alan Richmond, Allyn Damman, Camille Brown-Lowery, Princess Abbot-Grimes, Saira Siddiqui, Tigidankay Fadika, Mark Ward, Mia Cooper, Sonya Sutton, Lindsay Kenton, Bob Spaziano, Janet Kasper, Nicole Barnes, Christoph Hornik

**Affiliations:** 1 Duke Clinical Research Institute, Duke University School of Medicine, Durham, NC USA; 2 Duke Global Health Institute, Duke University School of Medicine, Durham, NC USA; 3 Department of Orthopaedic Surgery, Duke University School of Medicine, Durham, NC USA; 4 Community-Campus Partnerships for Health, Raleigh, NC USA; 5 United Way of Merced County, Merced, CA USA; 6 Pitt County Health Department, Greenville, NC USA; 7 Department of Pediatrics, Duke University School of Medicine, Durham, NC USA

**Keywords:** Community partners, COVID-19, Guidance, Health equity, Protocol, Public health, Toolkit, Underserved populations

## Abstract

**Background:**

Infections and deaths from the COVID-19 pandemic have disproportionately affected underserved populations. A community-engaged approach that supports decision making around safe COVID-19 practices is needed to promote equitable access to testing and treatment. You & Me: Test and Treat (YMTT) will evaluate a systematic and scalable community-engaged protocol that provides rapid access to COVID-19 at-home tests, education, guidance on next steps, and information on local resources to facilitate treatment in underserved populations.

**Methods:**

This direct-to-participant observational study will distribute at-home, self-administered, COVID-19 testing kits to people in designated communities. YMTT features a Public Health 3.0 framework and Toolkit prescribing a tiered approach to community engagement. We will partner with two large community organizations, Merced County United Way (Merced County, CA) and Pitt County Health Department (Pitt County, NC), who will coordinate up to 20 local partners to distribute 40,000 COVID tests and support enrollment, consenting, and data collection over a 15-month period. Participants will complete baseline questions about their demographics, experience with COVID-19 infection, and satisfaction with the distribution event. Community partners will also complete engagement surveys. In addition, participants will receive guidance on COVID-19 mitigation and health-promoting resources, and accessible and affordable therapeutics if they test positive for COVID-19. Data collection will be completed using a web-based platform that enables creation and management of electronic data capture forms. Implementation measures include evaluating 1) the Toolkit as a method to form community-academic partnerships for COVID-19 test access, 2) testing results, and 3) the efficacy of a YMTT protocol coupled with local resourcing to provide information on testing, guidance, treatment, and links to resources. Findings will be used to inform innovative methods to address community needs in public health research that foster cultural relevance, improve research quality, and promote health equity.

**Discussion:**

This work will promote access to COVID-19 testing and treatment for underserved populations by leveraging a community-engaged research toolkit. Future dissemination of the toolkit can support effective community-academic partnerships for health interventions in underserved settings.

**Trial registration:**

ClinicalTrials.gov Identifier: NCT05589376. Registered 21 October 2022.

**Supplementary Information:**

The online version contains supplementary material available at 10.1186/s12889-023-16960-6.

## Background

The COVID-19 pandemic decreased life expectancy and widened health and education disparities that will have a profound impact for generations [1]. In the United States, > 104 million people have been infected with the severe acute respiratory syndrome coronavirus 2 (SARS-CoV-2) virus, and > 1 million people have died [2]. Infections and deaths have disproportionately affected underserved populations who have been historically marginalized [3]. Inequitable risk of COVID-19 infection directly results from unequal access to care among racial and ethnic minority populations, including higher hospitalization rates, lower recovery rates, and higher mortality. Racial and ethnic minority populations also face social drivers of health, including unequal access to quality education, employment, and health care [3–5]. These inequities have exacerbated COVID-19 disparities, leading to loss of housing, health insurance, and food access in Black and Hispanic communities.

Public health interventions early in the pandemic focused on maximizing reach and rapidly distributing resources and knowledge to vulnerable communities. Two such interventions, Say Yes! COVID Test (SYCT) and You & Me COVID-Free (YMCF), examined ​​strategies to promote timely and equitable access to COVID-19 testing in support of decision making around safe COVID-19 practices [6, 7]. Both interventions provided antigen tests for at-home administration, education on testing and prevention, assessment of participant preference for accessing therapeutics at a doctor’s office versus pharmacy or other community setting, and an opportunity for remote participation in survey-based studies assessing behaviors around testing and COVID-19 prevention. These studies demonstrated that early involvement of community partners, clear and culturally appropriate messaging to participants, and respecting privacy by minimizing intrusiveness of data collection were essential to gain community and participant trust [6, 7]. SYCT and YMCF also showed that partnering with local organizations through scalable community-engaged strategies was crucial to reduce COVID-19 disparities related to timely testing and treatment access. Specifically, a tension exists when there is a rapid need for health interventions (such as with providing equitable access to COVID-19 testing) yet trusted relationships with community partners critical for effectiveness have not been established. Only through well-established community involvement, alignment, and mutual respect can community–academic research partnerships succeed to address health disparities [8]. Innovative ways to respond to community concerns in public health research are needed to foster cultural relevance, improve research quality, and promote health equity.

### Toolkit for engaging diverse communities to plan and implement public health programs

In response to this need, we developed a Community Engagement Toolkit for researchers, academics, public health agencies, and community partners to collaborate effectively on timely interventions aimed at reducing health disparities in underserved communities. The toolkit offers guidance for establishing community partnerships to plan and implement public health initiatives. The toolkit also addresses the need for innovation in community–academic research partnerships by recommending a tiered approach that involves working with an anchor, or large community partner that has established trusted relationships with smaller, local community-based organizations and with the community to ensure the intervention is meaningful and relevant. Community partners were co-creators of the toolkit, resulting in content that considers differences in drivers of health to promote equitable access to programs and services, and tailoring that is community specific.

The toolkit was designed based on a Public Health 3.0 framework. As outlined at the core of Public Health 3.0 (see Table 1) [3, 4, 6, 7, 9, 10], local communities should lead the charge in taking public health to the next level [9]. Grounded in this approach is the acknowledgement that social and structural factors are key drivers of health inequalities and that these inequalities cannot be addressed without the wisdom and expertise of community stakeholders. Stakeholders bring deep knowledge of the historical trauma, lived experiences, values, and concerns of the community that underline the health inequities and are necessary to consider when developing solutions. As a result, bidirectional capacity-building, co-learning, resource sharing, and communication between community and academic partners are critical to address health disparities. Under this premise, partnerships between academia and community are essential to equip local communities with the knowledge, tools, and expertise to design, implement, and disseminate interventions. The benefits of engaging communities have been reported in all stages of research including 1) identifying key research questions based on firsthand knowledge and insight from the field; 2) designing informed consent processes and research protocols that meet the needs of the community; 3) adapting interventions to be context-relevant; 4) identifying implementation processes to promote intervention feasibility and adoption; and 5) disseminating research results to make information more accessible [8, 11, 12]. Subscribing to a Public Health 3.0 framework, therefore, entails community members serving as authentic partners in research, and both community and research resources are leveraged to address social, environmental, and economic conditions that magnify health disparities in communities [13].

**Table 1:** Defining components of community engagement in the context of You & Me: Test and Treat (YMTT)

Term	Definition in Context of YMTT
Health Equity	Focus on promoting access to COVID-19 testing and treatment for underserved and vulnerable populations defined geographically with a high proportion of Hispanic/Latinos, Blacks, and socioeconomically disadvantaged participants. Health care has been historically plagued with discrimination, power inequities, and differential access to resources [3, 4]
Public Health 3.0	Apply the Public Health 3.0 framework [9] of collaboration among health care researchers (academia) and non-traditional community partners, specifically community leaders and trusted representatives, community organizations, and local stakeholders to strengthen the current research infrastructure
Trust	Build on preexisting relationships with community partners [6, 7] and tailor community engagement strategies to ensure access for the communities served. This is applied in YMTT as we will team up with community partners whom we previously worked with through Say Yes! COVID Test (SYCT) and You & Me COVID-Free (YMCF)
Precision Public Health	Utilize a precision public health approach [10] that relies on the trust, knowledge, and expertise of the local community partners to create tailored approaches to COVID-19 test distribution and treatment access. This principle can be seen within YMTT as participants give input on where they want access to support and other resources, and our team works to link them to the resources

### You & Me: Test and Treat

The You & Me: Test and Treat (YMTT) intervention utilizes the Community Engagement Toolkit with the goal of promoting COVID-19 test and treatment access in underserved communities. This project is funded by the National Institutes of Health (NIH)-led Rapid Acceleration of Diagnostics–Underserved Populations (RADx-UP) program, which is a consortium dedicated to understanding factors that led to the disproportionate burden of the pandemic on underserved populations and implementing interventions to mitigate these disparities [14]. The aims of YMTT are to 1) create effective academic and community research partnerships within Pitt County, NC, and Merced County, CA; 2) collect the results of the at-home tests we will distribute; and 3) evaluate this test-to-treat model and its ability to be customized per the recommendations of community partners. Building on the SYCT and YMCF studies, YMTT will develop testing and treatment access strategies together with trusted community organizations in high-need areas. We identified communities using risk assessment strategies to address barriers to test access, determine infrastructure needs, and prioritize timely testing access, awareness, education, and engagement with health systems and local resources. Additionally, YMTT uses Colectiv, a web-based data collection platform that enables creation and management of electronic consenting and data capture forms. Surveys submitted by participants via the app will be used to determine health topics of interest within the community, and a newsletter will be sent out to participants addressing these topics. Additionally, YMTT provides linkages (URLs and direct contact) to community resources if participants test positive. Ultimately, the YMTT intervention aims to reduce COVID-19 related health disparities through a tiered community-engaged approach to test and treatment access.

YMTT anchor partners were selected in both counties based on a history of successful partnerships in YMCF and SYCT. Anchor and community partners will distribute COVID-19 test kits during community events and advertise the study in their communities. Via the YMTT website, participants will be given access to guidance and care if they test positive or have questions. Local pharmacies and other trusted community sites will be identified by anchor partners to provide COVID-19 therapeutics and other resource linkages in response to a positive reported test to ensure timely notification and access to treatment.

Merced and Pitt counties were selected based on disproportionately high prevalence of Hispanic/Latino/Latinx (63% in Merced County) and Black (37% in Pitt County) populations living in poverty (22% for both counties) [15]. The communities also were chosen based on higher-than-average lack of health insurance (11% and 12% for Merced and Pitt Counties, respectively; national average: 8.5%) and higher county-level COVID-19 risk based on the Pandemic Vulnerability Index (Pitt County: 0.60; Merced County: 0.55, index range 0–1). As outlined above, we have previously conducted community-engaged test distribution and knowledge dissemination projects in both communities (Merced County: YMCF; Pitt County: SYCT) and established robust community partnerships.

## Methods

### The Community Engagement Toolkit overview

YMTT provides a case study, putting the recommendations of the toolkit in practice in both Merced County and Pitt County. The toolkit (publicly available at https://youandmehealthy.org/resources/) provides concrete strategies to address study needs across planning, implementation, study completion, and beyond [16]. The toolkit is divided into sections that address 1) community engagement, 2) public program design and planning, 3) communications and marketing, 4) operations and logistics, and 5) data collection and reporting. The toolkit utilizes images, diagrams, tools/templates, and case examples from SYCT and YMCF in its instruction. The YMTT study will apply all of the toolkit components (see Table 2), from design and implementation to dissemination.

**Table 2:** Topics addressed and tools provided in the Community Engagement Toolkit

Toolkit Components	Description
Community Engagement	Working *with* a community rather than working *in* a community
Public program design and planning	Orientation of community partners, distributing supplies, and gathering feedback on the progress and structure of the intervention
Communications and marketing	Maintaining relationships with partners and publicizing events
Operations and logistics	Running program sites and events
Data collection and reporting	Gathering and organizing data and distributing results to participants
Tools provided	Tips, templates, and case study examples from previous work on two public health programs (SYCT, YMCF), including information on how to build a leadership team, designate key member roles, develop partnerships, establish metrics, and address data needs, along with links to related resources

*Community engagement*. The toolkit includes educational graphics on community engagement that relay the benefits of this practice, including enhancing social capital, learning from program partners, and reaching the right people. Additionally, resources to learn about further methods and principles of community engagement are provided.

*Public program design and planning* includes information on how to find a lead community partner, foster and create teamwork, develop partnerships, and provide compensation. Necessary approvals for public health work, including institutional review board, approvals for events, and insurance requirements are included. This ensures that the rights and welfare of all people in research are protected, and that events are well organized and responsive to community needs. In addition, case examples from SYCT and YMCF are included that detail the essential role community partners played in the program design.

**Fig. 1:**
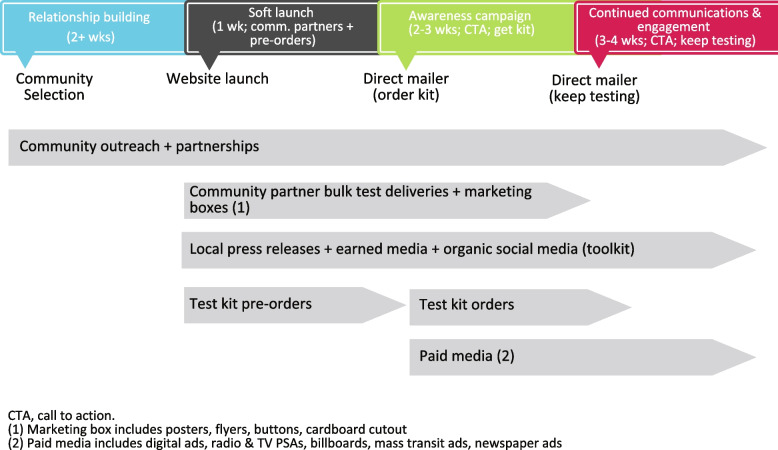
Sample timeline for distribution of communication tools from past studies included along with a more extensive template available within the Toolkit (see Communications Plan for Say Yes! COVID Test in Additional file 1)

The *communications and marketing* section of the toolkit includes information on setting up social media, announcing the launch of the program, and generally spreading the word in the community (see Fig. 1). Templates from SYCT and YMCF are included, such as a communications plan, sample print materials, sample key messaging framework, and a guide to communications channels.

The *operations and logistics* section focuses on managing program supplies. The toolkit also contains case examples from YMCF and SYCT on problems that might arise and how they were dealt with in these projects.

**Fig. 2:**
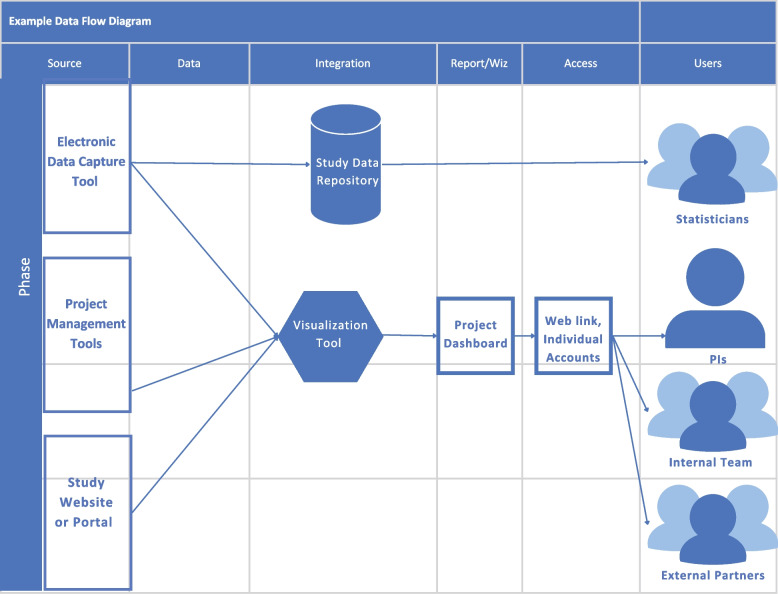
Example data flow diagram included as a resource within the Toolkit

**Fig. 3:**
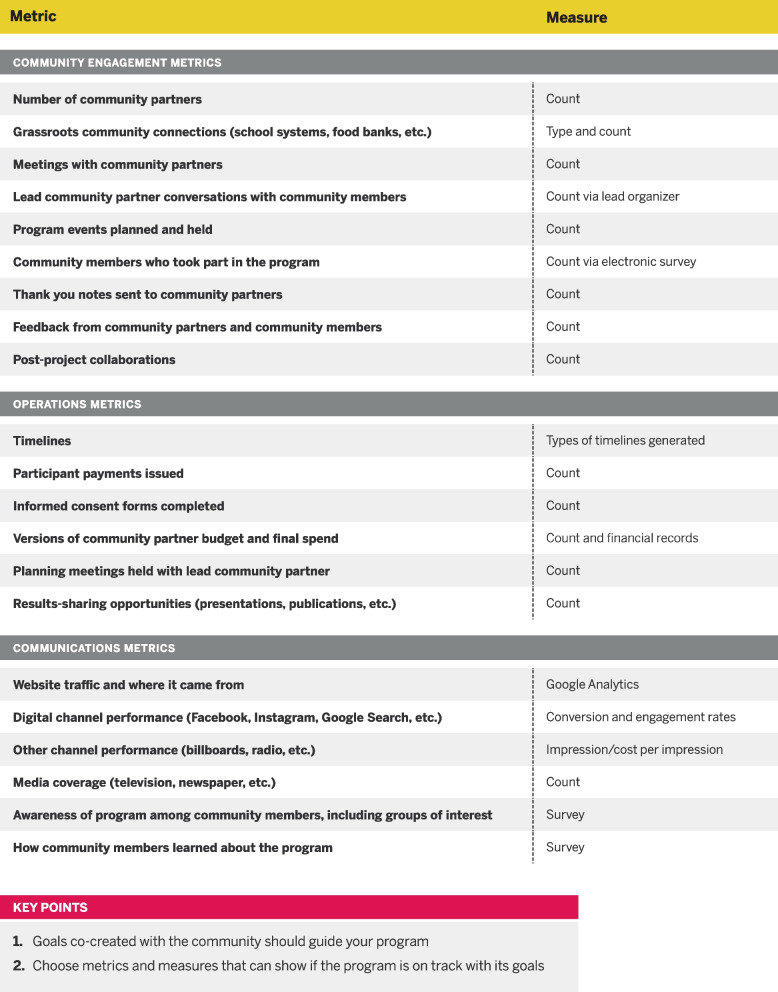
Guide of possible metrics and measures to use in community engagement studies

*Data collection and reporting* discusses surveys and data systems. The data collection system used for SYCT and YMCF is shown as an example, and the thought process behind this system is explained (see Fig. 2). Additionally, the toolkit contains a detailed chart (Fig. 3) that demonstrates metrics and measures from interventions within the areas of community engagement, operations, and communications.

### Study design

This study will use a tiered approach (working together with anchor partners who liaise with local community organizations) to distribute COVID-19 tests in Merced County and Pitt County. This protocol draws from HIV detection and treatment interventions [13, 17], and combines community engagement strategies developed through the toolkit. Two anchor partners (Merced County United Way and Pitt County Health Department) will each coordinate directly with up to 10 local partners to distribute 20,000 COVID test kits (40,000 total tests for the study) at community-based testing events (location and timing based on community partner guidance) over a 15-month period. All eligible consented participants at testing events will, using the Colectiv app, complete basic data collection including demographics, COVID-19 testing and treatment questions, additional testing satisfaction survey questions, and contact information. Community partners will also complete feedback surveys using the Colectiv app. All participants will receive compensation. Additionally, participants will receive guidance on COVID-19 mitigation, links to health-promoting resources, and accessible and affordable therapeutics if they test positive for COVID-19. We will leverage the You and Me Healthy Direct-to-Participant Registry (YMH registry) to provide resource linkages to trusted health services in local areas as identified by anchor institutions. The YMH registry provides ongoing notification to participants that they are eligible for enrollment in new research studies related to COVID-19, health equity, and reducing health disparities for underserved populations. For individuals without insurance, participants will be connected to local resources including mobile health clinics (typically serving under- and uninsured individuals) and community health centers. Additional local resources, such as pharmacies, fire departments, and churches offering free testing and treatment, will be identified by our anchor partners.

The protocol will be tailorable to each community but will address the following key elements: testing frequency, regular cadence of distribution of education materials, equitable access to treatment resources when appropriate, timely access to treatment resources when appropriate, proportion of positive cases provided with education resources on “next steps” to mitigate spread, change in participant awareness of COVID-19 mitigation strategies and treatment options, and proportion of positive cases provided with linkage to a community testing site, such as a pharmacy, clinic, or mobile van.

### Settings, participants and recruitment

The study will be conducted in underserved populations in Merced County and Pitt County (Table 2). Participants must have a self-reported primary residence within the intervention communities and be ≥ 8 years old when they receive the tests. Participants ≤ 14 years old will be instructed to have testing performed on them by an adult as per the Food and Drug Administration Emergency Use Authorization (authorized March 1, 2021) of the over-the-counter Quidel Quickvue At-Home COVID-19 test. Participants will be recruited for the study at test kit distribution events by community partners. This may include social events associated with high risk of infection (e.g., indoor festivals or concerts) or higher risk population (e.g., the elderly).

### Intervention

We will utilize a hybrid virtual and in-person recruitment process. Targeted media, web, and social media campaigns will inform potential participants of test kit distribution events by community partners. Participants will also be informed about the YMH registry through the YMTT newsletter. Participants in the YMH registry will be offered participation in YMTT, and the registry’s direct-to-participant engagement structure will be leveraged to recruit and retain participants in this study. The YMH registry can support our study in the following ways: 1) by adding YMTT as an appendix to the registry protocol to streamline review and approval; 2) by offering participation in YMTT to registry participants; 3) by capturing data needed for the YMTT protocol, including the collection of NIH common data elements [18], via the registry platform (Colectiv); and 4) by serving as a mechanism for recruitment and dissemination of results directly to participants.

### Outcome measures

A primary outcome of this work is to evaluate the Community Engagement Toolkit as a method to form community–academic partnerships for COVID-19 test access. In addition, results of COVID-19 at-home testing can be documented through the Colectiv platform. This work will also reflect the efficacy of a YMTT protocol coupled with local resourcing to provide information on testing, guidance, treatment, and link to resources.

Secondarily, this work will allow for the evaluation of the quality and quantity of community engagement. We will also be able to measure participant satisfaction with testing events, which will allow us to make modifications for the future.

### Analysis

We will conduct a comprehensive summary of all collected data including basic descriptives on demographics, test results, treatment, and beliefs (regarding vaccination and treatment), and associations between test results and behavioral measures and access to treatment will be explored using mixed modeling techniques to account for within-participant correlations. Analyses will be conducted using SAS 9.4 (SAS Institute, Cary NC).

We will report the total number of tests distributed and summarize participant characteristics using standard summary statistics and graphical techniques. The number of disseminated tests and consented participants by community, anchor partner, local partners, and specific distribution events or locations will be provided. We will also report the proportion (with 95% exact confidence intervals) of positive tests. Data may be stratified by participant and community partner characteristics and by distribution event. In addition, we will report 1) the proportion of participants who are provided with information on next steps for COVID-19 mitigation and information on accessible treatment locations (as determined by the anchor partner in collaboration with local community partners) within 5 days of reporting a positive test result, and 2) self-reported treatment utilization (type, duration). Lastly, we will conduct hypothesis testing and multivariable modeling using appropriate methods (e.g., mixed logistic and Poisson regression) to explore adjusted relationships between community/participant characteristics and outcomes (test positivity, behaviors, and care access). We will report regression coefficients when models meet assumptions and fit.

### Dissemination strategy

Because the Colectiv platform allows for real-time aggregate review of data, we will provide our participants with frequent aggregate data summaries that highlight study progress. Once our analysis is completed, we will prepare results in lay-friendly formats for dissemination to maximize public health impact. These will be shared through the YMTT and RADx-UP Coordination and Data Collection Center websites and social media channels, and returned directly to participants via the YMTT participant newsletter.

## Discussion

Community-engaged research relies on authentic and robust partnerships [19]. Collaborations across academic and community organizations are critical to promoting equitable access to health care for underserved communities. However, establishing these partnerships requires copious time, ample resources, and consistent investment from all partners to achieve success. Academics and other researchers can offer helpful expertise and skillsets, although they likely would not have established the necessary partnerships with local organizations and key stakeholders for those interventions to be effective. The Community Engagement Toolkit presents a tiered model for supporting timely interventions in underserved communities in response to widespread health events. The toolkit leverages anchor institutions that have trusted relationships already established with local community partners, who in turn are trusted by the communities that they serve. Through this approach, YMTT offers a case study for community-engaged health disparities research that is effective, timely, and consistent with the need for academic partners to support and follow the lead of local community organizations and stakeholders to effect change.

Adopting this tiered model for community-engaged research, the YMTT study has enrolled 684 participants to date (66.3% female; 72.4% White, 4.3% American Indian or Alaska Native, 3.8% Black, 1.3% Asian; 93.3% Hispanic; 75.4% adults; 59.6% English language preference) from March 24, 2023, through July 3, 2023 (see Table 3).

**Table 3:** Demographics and other characteristics for YMTT survey participants (*N* = 684)

Characteristic	n (%)
Age, years (*n* = 684)	
≥ 18	516 (75.4)
< 18	168 (24.6)
Gender identity term (*n* = 584)	
Woman	387 (66.3)
Man	194 (33.2)
Other^a^	3 (0.5)
Race^b^ (*n* = 373)	
White	270 (72.4)
Other/multiple	67 (16.9)
American Indian or Alaska Native	16 (4.3)
Black or African American	14 (3.8)
Asian	5 (1.3)
Native Hawaiian or Other Pacific Islander	1 (0.3)
Ethnicity (*n* = 537)	
Hispanic/Latino/Spanish	501 (93.3)
Not Hispanic/Latino/Spanish	36 (6.7)
Language of survey completed^c^	
English	408 (59.6)
Spanish	276 (40.4)
Where they heard about test kit distribution^b^ (*n* = 575)	
A community organization	517 (89.9)
Word of mouth	44 (7.7)
Social media	14 (2.4)
Other	12 (2.1)
Flyer	11 (1.9)
Community bulletin board	10 (1.7)
Total number of zip codes represented (*n* = 575)	38

^a^Includes “gender non-binary/genderqueer/gender nonconforming,” “two-spirit,” and “none of these describe me.”

^b^Participants could indicate all answer choices that applied

^c^Participants could select whether to complete the survey in English or Spanish

Participants on average have rated the experience of acquiring the COVID-19 test kit as 9.2 on a 1–10-point scale, with 10 being the highest satisfaction with the experience. YMTT also has held biweekly anchor and community partner meetings to support implementation, troubleshoot challenges with recruitment and data collection, and share best practices. Routine and open dialogue with the community partners also led to the development of narrated videos for both community partners and participants to support enrollment and use of the Colectiv app, specific newsletter content, anchor partner–led trainings for local partners on study enrollment, and tailoring communications materials to provide simpler and more direct messaging.

The YMTT study has strategized to address several challenges. To begin, Colectiv is a new platform established in 2021 with advanced data collection and potential for bidirectional communication functionalities that has required extra time to ensure accuracy and rigor. These delays introduced the need for additional support and communication with community partner organizations during intervention rollout to hold testing events and enroll participants at the events. The project also encountered turnover in anchor partner leadership and community partner staff in one of the project counties, resulting in challenges onboarding community partners and enrolling participants in those communities. In addition, the project is faced with overall “COVID-19 fatigue,” namely that participants are less motivated to attend events and enroll in a study that offers as its main incentive free COVID-19 testing and facilitated access to treatment. In addition, working remotely to lead a project in two communities presents challenges related to differences in time zones (to arrange team meetings), weather (such as major flooding in Merced County during partner onboarding), and communication (e.g., some partners preferred materials translated in languages not anticipated, such as Hmong, resulting in delays due to translation and institutional review board approval). These challenges are not atypical for community-engaged research [20], although the toolkit was a critical resource in addressing many of them.

**Fig. 4:**
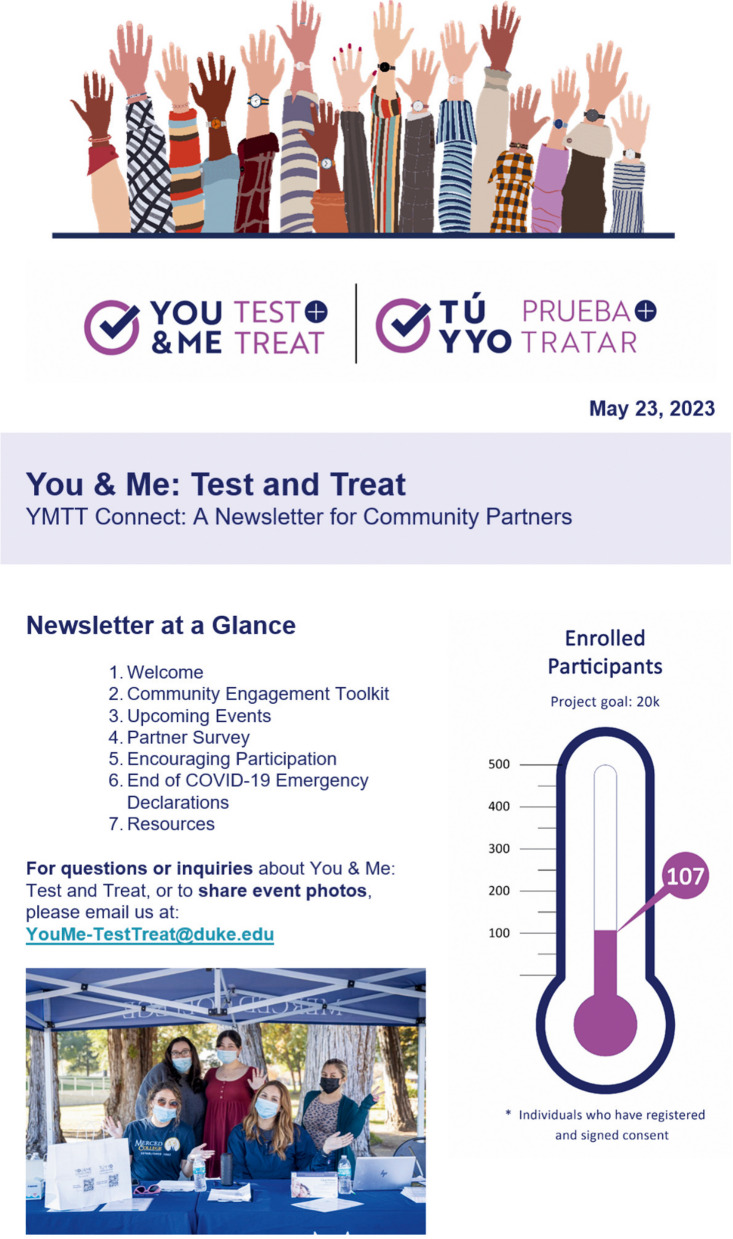
Excerpt from sample You & Me: Test and Treat (YMTT) newsletter for community partners

Next steps for YMTT include continuing to offer enrollment and test kits at community partner–led events in both Merced and Pitt counties, distributing community partner email newsletters (see Fig. 4 for an excerpt from the first YMTT newsletter for sample topics and featured quotes from SYCT and YMCF studies), and improved bidirectional communication with community partners and participants. Specifically, the, Colectiv app will be used to administer surveys to both community partners and participants in order to determine health topics of interest, testing and treatment needs, and other community member issues that can be addressed in part with resource linkages, newsletter information, and more tailored communication and dissemination materials.

## Conclusion

The YMTT study aims to reduce COVID-19 related health disparities through a community-engaged approach to test and treatment access. To achieve this goal, YMTT uses a tiered model of community-engaged research by leveraging large local organizations that are trusted by community partners and populations where timely health interventions are needed. Through this model, academic and other research organizations can effectively partner with community organizations to address health inequities. YMTT will provide evidence, using this tiered approach, to address COVID-19 testing and treatment disparities in two US counties. Future dissemination of YMTT findings, and the toolkit employed in this approach, will support effective and efficient community-engaged health interventions to close disparity gaps.

## Supplementary Information

**Additional file 1.** Say Yes! COVID Test Communications Plan (MS Word).

## Data Availability

The datasets used and/or analyzed during the current study are available from the corresponding author on reasonable request.
