# Correction: You & me: test and treat study protocol for promoting COVID-19 test and treatment access to underserved populations

**DOI:** 10.1186/s12889-024-20831-z

**Published:** 2025-03-06

**Authors:** Emily M. D’Agostino, Lauren M. Rosenberg, Alan Richmond, Allyn Damman, Camille Brown‑Lowery, Princess Abbot‑Grimes, Saira Siddiqui, Tigidankay Fadika, Mark Ward, Mia Cooper, Sonya Sutton, Lindsay Kenton, Bob Spaziano, Janet Kasper, Nicole Barnes, Christoph Hornik

**Affiliations:** 1 Duke Clinical Research Institute, Duke University School of Medicine, Durham, NC USA; 2 Duke Global Health Institute, Duke University School of Medicine, Durham, NC USA; 3 Department of Orthopaedic Surgery, Duke University School of Medicine, Durham, NC USA; 4 Community-Campus Partnerships for Health, Raleigh, NC USA; 5 United Way of Merced County, Merced, CA USA; 6 Pitt County Health Department, Greenville, NC USA; 7 Department of Pediatrics, Duke University School of Medicine, Durham, NC USA

**BMC Public Health (2023) 23:2121**

10.1186/s12889-023-16960-6

The original publication of this article contained incorrect trial registration information in the abstract. The incorrect and correct information are shown in this correction article. The original article has been updated.

Incorrect

ClinicalTrials.gov Identifier: NCT05455190. Registered 13 July 2022

Correct

ClinicalTrials.gov Identifier: NCT05589376. Registered 21 October 2022

