# Supplementary material for: You & Me: Test and Treat study protocol for promoting COVID-19 test and treatment access to underserved populations
Source: BMC Public Health. 2023 Oct 28;23:2121. doi: 10.1186/s12889-023-16960-6 (PMC10612258; doi:10.1186/s12889-023-16960-6)
Supplement: Supplementary file 1 — Additional file 1. Say Yes! COVID Test Communications Plan (MS Word). [file 12889_2023_16960_MOESM1_ESM.docx]

**TOOLS AND TEMPLATES**

**Example Communications Plan**

**
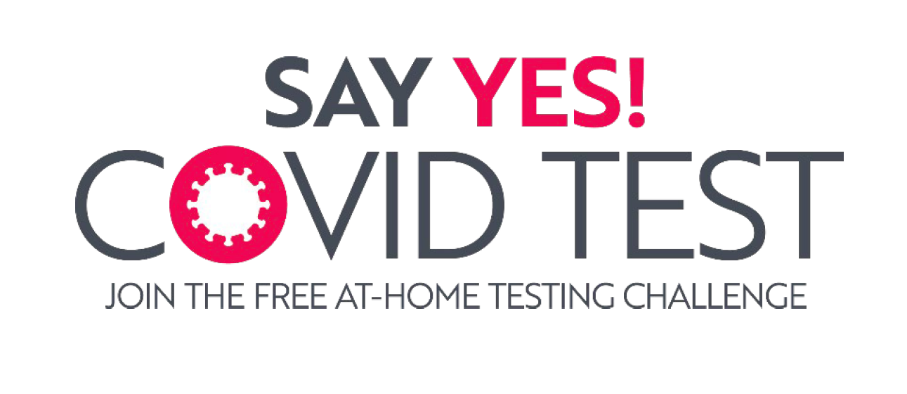
**

**Communications Plan**

Example for Community Engagement Toolkit

Table of Contents

[Say Yes! COVID Test Communications Plan 2](#_Toc65834754)

[Goal/Objectives 2](#_Toc65834755)

[Target Audiences 2](#_Toc65834756)

[Branding and Messaging 2](#_Toc65834757)

[Branding 3](#_Toc65834758)

[Key Messages 3](#_Toc65834759)

[Tone and Voice 3](#_Toc65834760)

[Communication Channels, Timing, Frequency, & Evaluation 4](#_Toc65834761)

[Community Partner Toolkit in a Box 9](#_Toc65834762)

# Communications Plan

## Goal/Objectives

Include overall goal of the communications plan and a list of objectives for the project.

The purpose of this communications plan is to XXXX

The public information campaign aims to achieve the following objectives:

- Objective
- Objective
- Objective

## Target Audiences

Include target audiences and any inclusion criteria for a program.

## Branding and Messaging

Consistent branding and messaging is important across all channels. Include here all the groups who will use the branding and logos, and how they can be used (print materials, digital, etc.). Include the exact way you’d like the project referred to here, including any capitalization and punctuation.

### Branding

Include program name and if there will be any customization for different communities or groups. See examples below:


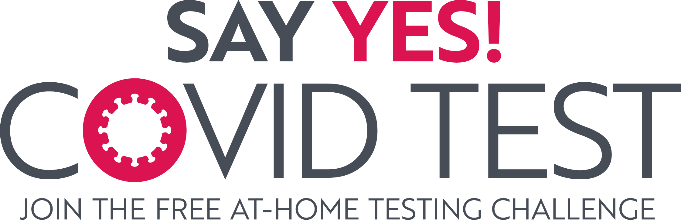


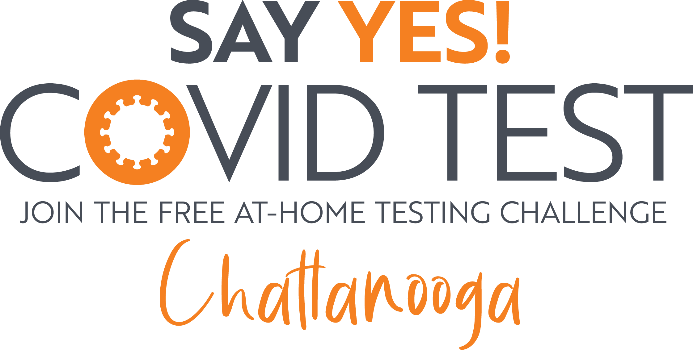


Provide guidance here on how to use the names in different occasions, including capitalization, punctuation, and whether or not to include information about the funder. If you are partnering with a commercial organization, find out what they need to review and approve and include here as well.

### Key Messages

What is the most important thing you want your audiences to know about the program? List those messages here.

Example:

The ultimate goal of the public information campaign and testing initiative is to reduce community spread of COVID-19. By providing free, rapid at-home tests for COVID-19, we are putting power in the hands of individuals to be able to keep themselves, their family, and their community safe. Please refer to the Key Messages Document for the full list of key messages.

### Tone and Voice

The public information campaign’s tone across channels should be:

- Clear
- Personal
- Empowering
- Motivating

The public information campaign’s voice is:

- Informative
- Action oriented
- Community focused and friendly
- Accessible
- Supportive
- Inclusive
- Concise

## Communication Channels, Timing, Frequency, & Evaluation

Please see the graphic below summarizing the key communication tools and examples from our program. A further explanation of key channels is outlined below the graphic. You can update these to reflect your channels and tools.


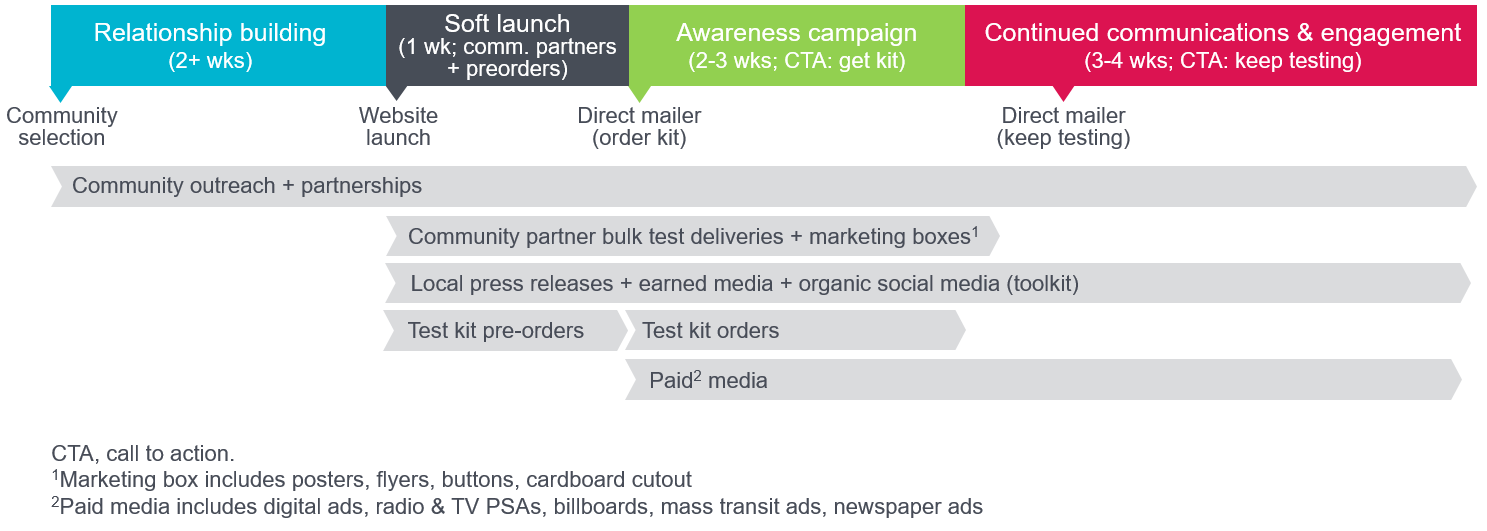


| Channel | Announcement to Local Press |
| --- | --- |
| Audience | Potential and current participants in the testing initiative and research study, community residents, community leaders, media |
| Community specific? | Yes |
| Spanish translation? | Yes |
| Purpose/Goal | - Promote media coverage of the initiative to spread awareness and excitement throughout the local community - Demonstrate community leader support for the initiative through quotes - Establish trust through a connection with the local health department - Encourage visiting the website for more information and to sign up for the at-home testing challenge (order your test kits) |
| Timing/Frequency | Once around March 29 (program launch) |
| Measurement | Number of stories run on local media, Say Yes! COVID Test website visits and other Google Analytics metrics (eg, time on site, bounce rate), number of test kit orders |

| Channel | Say Yes! COVID Test Community Website |
| --- | --- |
| Audience | Potential and current participants in the testing initiative and research study, community residents, community leaders, media |
| Community specific? | Yes |
| Spanish translation? | Yes |
| Purpose/Goal | - Serve as a central point of information for all testing initiative details - Facilitate test kit orders (through linkage to the CareEvolution site) or pick-up at local locations - Encourage community participation in the testing initiative and completion of the at-home testing challenge - Provide instructions on what to do with test results and link to CDC and local health resources - Introduce the optional research study opportunity - Provide lay summary results from the initiative once available |
| Timing/Frequency | The website will be the first communication channel to launch for the campaign and will remain live throughout the campaign and for a period of time afterwards. Content will need to be updated throughout the course of the campaign to transition the focus from test ordering to continued testing use. After completion of the campaign, the website can remain live to share initiative results with the community. |
| Measurement | Website visits and other Google Analytics metrics (eg, time on site, bounce rate), number of people directed to the test kit order site |

| Channel | Initial Direct Mailer Postcard |
| --- | --- |
| Audience | All households in selected community |
| Community specific? | Yes |
| Spanish translation? | Yes |
| Purpose/Goal | - Introduce the testing initiative - Provide a clear call to action - Encourage visiting the website for more information and to sign up for the at-home testing challenge (order your test kits) - Introduce the optional research study |
| Timing/Frequency | Once at start of campaign |
| Measurement | Number of postcards sent and delivered, Say Yes! COVID Test website visits and other Google Analytics metrics (eg, time on site, bounce rate), number of people directed to the test kit order site |

| Channel | Packing Slip With Test Kit (Inside Amazon Box) |
| --- | --- |
| Audience | Participants who have ordered a test kit online |
| Community specific? | No |
| Spanish translation? | No |
| Purpose/Goal | - Give clear, concise instructions on how to participate in the at-home testing challenge - Encourage visiting the Say Yes! COVID Test website for more information - Prompt use of the online digital assistant for testing support |
| Timing/Frequency | Once with test kit |
| Measurement | Say Yes! COVID Test website visits and other Google Analytics metrics (eg, time on site, bounce rate), number of people directed to the online digital assistant site |

| Channel | Instructional Postcard |
| --- | --- |
| Audience | Participants receiving a test kit |
| Community specific? | Yes |
| Spanish translation? | Yes |
| Purpose/Goal | - Give clear, concise instructions on how to participate in the at-home testing challenge - Provide a source for more information about the testing challenge - Introduce the optional research study opportunity |
| Timing/Frequency | Sent once via direct mail at the same time as kit order (sent separately) or handed out with test kit at pick-up site |
| Measurement | Postcards distributed, Say Yes! COVID Test website visits and other Google Analytics metrics (eg, time on site, bounce rate) |

| Channel | Reminder Direct Mail Postcard |
| --- | --- |
| Audience | Participants who have ordered a test kit online |
| Community specific? | Yes |
| Spanish translation? | Yes |
| Purpose/Goal | - Encourage continued use of testing kits according to campaign goals - Provide a source for more information should the participant have questions |
| Timing/Frequency | Sent once via direct mail ~2 weeks after test kit order |
| Measurement | Postcards distributed, Say Yes! COVID Test website visits and other Google Analytics metrics (eg, time on site, bounce rate) |

| Channel | Social Media Toolkit |
| --- | --- |
| Audience | Local public health departments, community leaders, and community partners (eg, schools, employers) |
| Community specific? | Yes |
| Spanish translation? | No |
| Purpose/Goal | - Provide community leaders and partners with ready-to-use content, graphics, and videos to post on their existing social media channels - Raise awareness of the testing initiative and encourage participation - Drive traffic to the campaign website for more information and to sign up - Remind participants to continue the at-home testing challenge throughout the campaign |
| Timing/Frequency | Distributed at the beginning of the campaign, with use encouraged throughout the campaign |
| Measurement | Use of specific hashtag(s), number of posts and shares, social media−driven Say Yes! COVID Test website visits and other Google Analytics metrics (eg, time on site, bounce rate), community feedback |

| Channel | SMS Text Reminders |
| --- | --- |
| Audience | Participants in the testing initiative who have signed up to receive text reminders |
| Community specific? | No |
| Spanish translation? | Yes |
| Purpose/Goal | - Provide test kit delivery status updates - Remind participants to continue the at-home testing challenge throughout the campaign - Give accurate and concise information at appropriate times |
| Timing/Frequency | From test kit order (SMS sign-up) through completion of the testing challenge |
| Measurement | Number of SMS sign-ups, number of SMS messages sent, number of opt-outs, community/user feedback |

| Channel | Digital Ads (Banner Ads, Social Media Ads, and Other Paid Online Ads) |
| --- | --- |
| Audience | Potential and current participants in the testing initiative and research study, community residents, community leaders |
| Community specific? | Yes |
| Spanish translation? | Yes |
| Purpose/Goal | - Introduce the testing initiative in a way that sparks interest, such as through appealing copy, animated graphics, etc. - Provide a clear call to action - Encourage visiting the website for more information and to sign up for the at-home testing challenge (order your test kits) |
| Timing/Frequency | From beginning of campaign through duration of campaign |
| Measurement | Number of ad clicks, referral Say Yes! COVID Test website visits and other Google Analytics metrics (eg, time on site, bounce rate) |

| Channel | Radio and TV Public Service Announcements |
| --- | --- |
| Audience | Potential and current participants in the testing initiative and research study, community residents, community leaders |
| Community specific? | Yes |
| Spanish translation? | Yes |
| Purpose/Goal | - Introduce the testing initiative in a way that sparks interest - Provide a clear call to action - Encourage visiting the website for more information and to sign up for the at-home testing challenge (order your test kits) - Remind participants to continue the at-home testing challenge throughout the campaign |
| Timing/Frequency | From beginning of campaign through duration of campaign |
| Measurement | Number of ads aired, Say Yes! COVID Test website visits and other Google Analytics metrics (eg, time on site, bounce rate) |

| Channel | Billboards and Mass Transit Ads (Buses, Bus Stops) |
| --- | --- |
| Audience | Potential participants in the testing initiative and research study, community residents, community leaders |
| Community specific? | Yes |
| Spanish translation? | No |
| Purpose/Goal | - Introduce the testing initiative in a way that sparks interest - Provide a clear call to action - Encourage visiting the website for more information and to sign up for the at-home testing challenge (order your test kits) |
| Timing/Frequency | From beginning of campaign through duration of campaign |
| Measurement | Number of ads posted, TBD |

| Channel | Newspaper Ads (Print) |
| --- | --- |
| Audience | Potential participants in the testing initiative and research study, community residents, community leaders |
| Community specific? | Yes |
| Spanish translation? | No |
| Purpose/Goal | - Introduce the testing initiative in a way that sparks interest, such as through appealing copy, animated graphics, etc. - Provide a clear call to action - Encourage visiting the website for more information and to sign up for the at-home testing challenge (order your test kits) |
| Timing/Frequency | TBD |
| Measurement | Number of ads printed, Say Yes! COVID Test website visits and other Google Analytics metrics (eg, time on site, bounce rate) |

| Channel | Earned Media (Radio, TV, etc) |
| --- | --- |
| Audience | Potential and current participants in the testing initiative and research study, community residents, community leaders |
| Community specific? | Yes |
| Spanish translation? | No |
| Purpose/Goal | - Establish media partnerships to earn coverage that can promote the campaign to the local community - Train local community spokespeople on key campaign messages - Encourage visiting the website for more information and to sign up for the at-home testing challenge (order your test kits) - Remind participants to continue the at-home testing challenge throughout the campaign |
| Timing/Frequency | Unknown |
| Measurement | Number of stories run, Say Yes! COVID Test website visits and other Google Analytics metrics (eg, time on site, bounce rate) |

| Channel | Town Hall Slides |
| --- | --- |
| Audience | Local public health departments, community leaders, and community partners (eg, schools, employers) |
| Community specific? | Yes |
| Spanish translation? | No |
| Purpose/Goal | - Provide community leaders and partners with ready-to-use slides they can insert into presentations they are giving - Raise awareness of the testing initiative and encourage participation - Drive traffic to the campaign website for more information and to sign up - Remind participants to continue the at-home testing challenge throughout the campaign |
| Timing/Frequency | Distributed at the beginning of the campaign, with use encouraged throughout the campaign. Also available for download from Say Yes! COVID Test website. |
| Measurement | Number of downloads from Say Yes! COVID Test website, community feedback |

### Community Partner Toolkit in a Box

The following items will be included in a box shipped separately with each bulk test kit order to facilitate community partner distribution of test kits. These items are intended to help advertise the testing initiative at the test kit pick-up site.

| Channel | Posters |
| --- | --- |
| Audience | Potential participants in the testing initiative and research study, community residents |
| Community specific? | Yes |
| Spanish translation? | Yes |
| Purpose/Goal | - Introduce the testing initiative in a way that sparks interest, such as through appealing copy - Provide a clear call to action to participate in the at-home testing challenge by ordering a test kit or picking up locally - Intended for use around high-traffic areas |
| Timing/Frequency | Distributed with each bulk testing kit order; also available for download from the website for self-printing |
| Measurement | Number of posters distributed and downloaded |

| Channel | Flyers |
| --- | --- |
| Audience | Potential participants in the testing initiative and research study, community residents |
| Community specific? | Yes |
| Spanish translation? | Yes |
| Purpose/Goal | - Introduce the testing initiative in a way that sparks interest, such as through appealing copy - Provide a clear call to action to participate in the at-home testing challenge by ordering a test kit or picking up locally - Intended to be handed out individually |
| Timing/Frequency | Distributed with each bulk testing kit order; also available for download from the website for self-printing |
| Measurement | Number of flyers distributed and downloaded |

| Channel | Face masks |
| --- | --- |
| Audience | Community leaders and distribution partners |
| Community specific? | Yes |
| Spanish translation? | No |
| Purpose/Goal | - Intended for community leaders and distribution partners to wear as a sign of support for the campaign |
| Timing/Frequency | Distributed with each bulk testing kit order |
| Measurement | Number of face masks distributed |

| Channel | Cardboard Floor Stands |
| --- | --- |
| Audience | Potential participants in the testing initiative and research study, community residents |
| Community specific? | Yes |
| Spanish translation? | No |
| Purpose/Goal | - Provide a clear call to action to participate in the at-home testing challenge by picking up a test kit |
| Timing/Frequency | Distributed with each bulk testing kit order |
| Measurement | Number of bulk test kits distributed |

| Channel | Tabletop Stands |
| --- | --- |
| Audience | Potential participants in the testing initiative and research study, community residents |
| Community specific? | Yes |
| Spanish translation? | No |
| Purpose/Goal | - Introduce the testing initiative in a way that sparks interest, such as through appealing copy - Provide a clear call to action to participate in the at-home testing challenge by ordering a test kit or picking up locally - Intended for use around high-traffic areas (eg, grocery store checkouts, break rooms) |
| Timing/Frequency | Distributed with each bulk testing kit order |
| Measurement | Number of stands distributed |
